# TIPS improves survival in patients with cirrhosis and recurrent ascites: a multicenter observational study

**DOI:** 10.1097/JS9.0000000000004579

**Published:** 2026-01-21

**Authors:** Bo Wang, Jun Zhu, Jing Li, Shoujie Zhao, Yejing Zhu, Shouzheng Ma, Liangzhi Wen, Enxin Wang, Tingting Bai, Dandan Han, Yan Zhao, Hui Chen, Yang Bai, Yanju Lou, Yongchao Zhang, Man Yang, Luo Zuo, Jiahao Fan, Xing Chen, Jia Jia, Wenbing Wu, Weirong Ren, Yuxin Tang, Dongfeng Chen, Guohong Han, Xilin Du, Chuangye He, Lei Liu

**Affiliations:** a Department of Infectious Diseases, Xijing Hospital, State Key Laboratory of Holistic Intergrative Management of Gastrointestinal Cancers and Xiing Hospital of Digestive Diseases, Fourth Military Medical University, Xi’an, China; b Department of General Surgery, Tangdu Hospital, Fourth Military Medical University, Xi’an, China; c Department of General Surgery, The Southern Theater Air Force Hospital, Guangzhou, China; d Department of Digestive Diseases, Shanxi Bethune Hospital, Shanxi Academy of Medical Science, Tongji Shanxi Hospital, Third Hospital, Shanxi Medical University, Taiyuan, China; e Tongji Hospital, Tongji Medical College, Huazhong University of Science and Technology, Wuhan, China; f Department of Thoracic Surgery, Tangdu Hospital, Fourth Military Medical University, Xi’an, China; g Department of Digestive Diseases, Daping Hospital, Third Military Medical University, Chongqing, China; h Department of Digestive Diseases, Air Force Hospital, Western Theater Command, Chengdu, China; i Department of Digestive Diseases, The First Affiliated Hospital of Xi’an Jiaotong University, Xi’an, China; j Department of Neurosurgery, General Hospital, Northern Theater Command, Shenyang, China; k Department of Orthopedic Surgery, Air Force Hospital, Western Theater Command, Chengdu, China; l Department of Medical Affairs, Air Force Hospital, Western Theater Command, Chengdu, China; m Center for Digestive Disease, The Seventh Affiliated Hospital of Sun Yat-sen University, Shenzhen, China; n Department of Digestive Diseases, The Second Affiliated Hospital of Chengdu Medical College, Chengdu, China; o Department of Digestive Diseases, The Affiliated Hospital of Southwest Medical University, Luzhou, China; p Department of Oncology, Qingdao Women and Children’s Hospital, Qingdao, China; q Department of Emergency, Shaanxi Provincial People’s Hospital, Xi’an, China; r Department of Digestive Diseases, Xi’an First Hospital, Xi’an, China; s Department of Digestive Diseases, Sanmenxia Central Hospital, Henan University of Science and Technology, Sanmenxia, China; t Department of Liver Diseases and Digestive Interventional Radiology, Digestive Diseases Hospital, Xi’an International Medical Center Hospital of Northwestern University, Xi’an, China; u Department of Interventional Vascular, Xi’an No.3 Hospital, The Affiliated Hospital of Northwest University, Xi’an, China

**Keywords:** cirrhosis, LVP + A, recurrent ascites, TIPS

## Abstract

**Background & Aims:**

Currently, there is limited evidence regarding the survival benefit of transjugular intrahepatic portosystemic shunt (TIPS) placement in patients with advanced cirrhosis and recurrent ascites. This study aimed to assess whether TIPS improves survival in such population compared with large-volume paracentesis plus albumin (LVP + A).

**Methods:**

This retrospective study included 462 patients with advanced cirrhosis and recurrent ascites who were treated with TIPS (*N* = 151) or LVP + A (*N* = 311) at 11 tertiary hospitals in China between September 2014 and September 2020. The Fine and Gray competing risk regression model was used to compare the outcomes between the two groups after adjusting for liver disease severity and other potential confounders.

**Results:**

The 1-year overall survival was significantly higher in the TIPS group than in the LVP + A group (77.9% vs. 47.3%; *P* < 0.001), with the relative risk of mortality reduced by 57% [adjusted hazard ratio (HR) = 0.43, 95% confidence interval (CI): 0.29–0.64; *P* < 0.001]. Furthermore, the TIPS group had a lower rate of further paracentesis (17.9% vs. 100%; *P* < 0.001) and portal hypertension (PHT)-related bleeding (5.8% vs. 28.1%; *P* < 0.001) but a higher rate of overt hepatic encephalopathy (OHE) (26.5% vs. 6.4%; *P* < 0.001) compared with the LVP + A group.

**Conclusions:**

In patients with advanced cirrhosis and recurrent ascites, TIPS improved the 1-year survival rates compared with LVP + A. Although TIPS increases the risk of OHE, it can significantly decrease ascites recurrence and PHT-related bleeding.

## Introduction

The development of refractory ascites (RA) is associated with a significant reduction in survival, reaching only 50% at 6 months. RA is linked to the development of many complications, including hyponatremia and progressive renal dysfunction[1]. The development of RA is accompanied by a poor prognosis and impaired quality of life in patients with cirrhosis^[2,3]^. Earlier guidelines recommended that RA management should follow a stepwise approach, including dietary sodium restriction, large-volume paracentesis plus albumin (LVP + A), or transjugular intrahepatic portosystemic shunt (TIPS) insertion in suitable patients, and consideration of liver transplantation (LT)[4].

LVP + A is a safe treatment option for patients with RA and provides immediate relief from symptoms[5]. Nevertheless, this therapy may have negative effects on systemic haemodynamics and renal function, whereas TIPS may improve renal function by ameliorating effective arterial blood volume and renal perfusion^[6, 7]^. Most studies evaluating the role of TIPS in RA were conducted before the introduction of expanded polytetrafluoroethylene (ePTFE)-covered stents^[8–13]^. Meta-analyses of these trials showed that TIPS was more effective in controlling ascites but was associated with an increased risk of severe hepatic encephalopathy (HE)^[13–19]^. Nevertheless, a recent randomized controlled trial (RCT) by Bureau *et al* challenged these results. The study showed that patients with recurrent ascites treated with covered TIPS had significantly higher 1-year transplant-free survival (TFS) than those treated with LVP + A (93% vs. 52%), without an increased incidence of HE[20]. To date, this study was the only RCT using ePTFE-covered stents and included patients who did not meet the strict definition of RA. The study included patients with recurrent tense ascites, defined as necessitating two LVPs in intervals of at least 3 weeks. Patients who required more than six LVPs within the previous 3 months were excluded. The study demonstrated a survival benefit of TIPS in patients with less severe cirrhosis/ascites and fewer paracentesis procedures, even if they did not meet the strict criteria for RA. Consequently, the European Association for the Study of the Liver (EASL) Clinical Practice Guidelines for decompensated cirrhosis recommend the use of TIPS early in the course of patients with ascites, emphasizing not to wait until patients develop severe liver dysfunction[21].

However, approximately half of the patients considered for the RCT by Bureau *et al* had to be excluded, mainly due to factors such as age > 70 years, Child–Pugh score >12 points, or requirement of >6 LVPs in the previous 3 months. These characteristics should be considered when considering TIPS for the management of ascites. Although optimal outcomes could be achieved with the careful selection of candidates, patients who do not meet these criteria may benefit from TIPS. Studies designed to evaluate whether less strict criteria could be used or whether patients with advanced RA could benefit from TIPS. Furthermore, these results need to be validated in real-world clinical practice because of the small sample size in the Bureau *et al*’s study. In addition, because most patients have alcoholic liver disease, whether the results could be generalized to other chronic liver diseases remains unknown.

This large cohort study aimed to evaluate whether TIPS could improve the survival of patients with advanced cirrhosis and recurrent ascites compared with LVP + A in real-world clinical practice.

**HIGHLIGHTS**

- Long-term longitudinal follow-up and real-world study.
- Transjugular intrahepatic portosystemic shunt (TIPS) improves transplant-free survival in patients with cirrhosis and recurrent ascites, even in different risk stratifications.
- TIPS may be beneficial to the survival of patients with ascites after the change of serum creatinine level 3 months later.

## Patients and methods

### Patients

This retrospective study extracted the clinical data of patients with liver cirrhosis who were hospitalized for recurrent ascites at 11 tertiary hospitals in China between September 2014 and September 2020. All participating centers had Gastroenterology/Hepatology Departments and experienced clinicians performing TIPS. According to institutional requirements, all patients provided written informed consent for each procedure before starting treatment.

The inclusion criteria were as follows: (1) a definite diagnosis of cirrhosis (based on liver biopsy or clinical symptoms, laboratory, and imaging studies), (2) recurrent ascites. According to the criteria of the International Ascites Club, recurrent ascites is defined as “ascites recurs on at least three occasions within a 12-month period, despite the prescription of dietary sodium restriction and adequate diuretic dosage”^[5, 22]^. (3) TIPS or LVP + A therapy, and (4) age >18 years. The exclusion criteria were as follows: (1) malignant neoplasm, including hepatocellular carcinoma with extrahepatic metastasis; (2) prior LT; (3) severe heart and lung dysfunction; (4) recurrent HE; (5) concomitant active infection; (6) complete portal vein thrombosis; (7) uncovered stents; (8) advanced extrahepatic malignancy; (9) a diagnosis of RA. Patients who received TIPS for recurrent ascites were classified into the TIPS group, and those who received standard treatment were classified into the LVP + A group. The work has been reported in line with the STROCSS criteria[23].

### Endpoints

The primary endpoints of the study were 1-year all-cause mortality. The secondary endpoints were recurrence of ascites (defined as persistent ascites volume requiring puncture despite the use of diuretics), development of overt HE (OHE; diagnosed and graded according to the West Haven criteria)[24], and uncontrolled portal hypertension (PHT)-associated bleeding.

### Therapeutic interventions

Patients were managed according to the Baveno V consensus workshops and American Association for the Study of Liver Diseases guidelines. The decision to institute therapeutic modifications, especially regarding placement of TIPS, was based on individual center policy and the judgment of local physicians according to their clinical assessment of the patients.

TIPS was performed by experienced clinicians in the Gastroenterology/Hepatology Department according to standard operating procedures, utilizing 8 or 10 mm PTFE-covered stents at the discretion of the local physician. LVP + A was performed as needed by the patient, the average interval between each paracentesis was 30 days (interquartile range: 15–45 days), the average volume of ascites extracted for each paracentesis was 5 L (interquartile range: 4–6 L), and when > 3 L of ascitic fluid was withdrawn, 8 g of albumin was infused intravenously per litre of ascitic fluid. All patients were followed at 1, 3, and 6 months and every 6 months thereafter. The revisits typically included routine laboratory examinations, abdominal B-ultrasound, computed tomography (CT), and other imaging examinations to assess the alleviation of ascites and identify any stent dysfunction. When patients underwent TIPS and developed complications related to PHT or dysfunction on Doppler ultrasonography, TIPS stent revision or new stent placement was performed. The follow-up time was defined as the interval between admission to the hospital and death, LT, last visit, or end of the study (18 September 2020).

### Stratification systems

We stratified patients to assess the impact of TIPS versus LVP + A on outcomes according to the following classification rules: Model of end-stage liver disease (MELD) 11–19 criteria (low risk/medium risk/high risk: MELD ≤11/11–19/ ≥ 19)[25], Child–Pugh class (low risk/high risk: Child–Pugh B/C)[26] and the Freiburg index of post-TIPS survival (FIPS)[27].

### Statistical analysis

Data are expressed as means ± standard deviations or numbers (%). Differences among continuous and categorical variables were examined for significance using Student’s *t*-test (or the Mann–Whitney *U* test) and chi-squared test (or Fisher’s exact test). The cumulative incidences of recurrent ascites, new OHE, and failure to control bleeding or rebleeding were estimated in a competitive risk environment in which death or LT competed with prognostic events. The Cox proportional hazards model was used for univariate and multivariate analyses. We tested the interaction between treatment and risk categories on both multiplicative (HR) and additive (absolute difference) scales.

For all analyses, the percentage of missing covariate values was <6%. Missing covariates were imputed using multiple imputations[28]. To verify the robustness of our results, two different sensitivity analyses with case-wise deletion of missing values and propensity score matching (PSM) were performed. The propensity score (PS) was generated using a logistic regression model, which included the following variables: patient age, HBV-DNA/HCV-RNA positivity at admission, bilirubin, international normalized ratio, albumin, creatinine, and platelet count. All analyses were conducted using BM SPSS Statistics for Windows, version 27.0. (IBM Corp., Armonk, NY, USA) and R (V.4.3.1, http://cran.r-project.org/). Two-sided *P* values <0.05 were considered statistically significant.

## Results

This study enrolled 1154 consecutive patients with cirrhosis and ascites admitted to 11 participating hospitals. Among them, 462 patients with recurrent ascites were eligible for the study. The disposition of patients is shown in the flowchart in online Supplemental Digital Content Figure 1, available at: http://links.lww.com/JS9/G475. Of the eligible patients, 151 patients (32.7%) received TIPS (TIPS group), and 311 patients (67.3%) received standard care (LVP + A group). Table 1 summarizes the baseline characteristics of the patients. The TIPS group had better liver function reserve, as reflected by lower Child–Pugh and MELD scores. There was significant difference in the use of furosemide (*P* < 0.001) and spironolactone (*P* < 0.001) between the two groups. The mean age of patients in the TIPS group was 51 years, with 108 men (71.5%). Stents of 8 and 10 mm were used in 119 (78.8%) and 32 (21.2%) cases, respectively. TIPS insertion with a decrease of mean portal vein pressure gradient from 24.9 ± 4.8 to 8.9 ± 2.8 mmHg (*P* < 0.001). The mean age of the patients in the LVP + A group was 56 years, with 207 men (66.6%). For 236 patients (51.1%) with a history of previous variceal bleeding, 35 patients (13.4%) were treated with β-blockers. Additionally, 277 patients (60.0%) had chronic HBV infection as the main cause, 32 patients (6.9%) had chronic HCV infection, 47 patients (10.2%) had alcoholic cirrhosis, 63 patients (24.0%) had miscellaneous cause, and 43 patients (16.4%) had other causes. All 302 patients (65.4%) with detectable HBV/HCV DNA levels received antiviral treatment, and a virological response was achieved in 256 (84.8%) patients at follow-up (80 in the TIPS group and 176 in the LVP + A group).

**Table 1:** Comparison of baseline characteristics between the two groups.

	TIPS group (*N* = 151)	LVP + A group (*N* = 311)	*P*
Age (years)	51.0 (44.0–62.5)	56.0 (47.0–67.0)	0.002
Sex	–	–	0.283
Male	108 (71.5)	207 (66.6)	–
Female	43 (28.5)	104 (33.4)	–
Etiology of cirrhosis	**–**	**–**	0.319
Chronic HBV infection	92 (60.9)	185 (59.5)	–
Chronic HCV infection	12 (7.9)	20 (6.4)	–
Alcoholic liver disease	16 (10.6)	31 (10.0)	–
Miscellaneous	23 (15.2)	40 (12.9)	–
Other	8 (5.4)	35 (11.2)	–
HBV-DNA detectable	64 (42.4)	152 (48.9)	0.190
HCV-DNA detectable	6 (4.0)	12 (3.9)	0.952
Previous variceal bleeding	72 (47.7)	164 (52.7)	0.308
Previous treatment with β-blockers	10 (6.6)	25 (8.0)	0.590
Encephalopathy	8 (5.3)	15 (4.8)	0.826
Hepatorenal syndrome	5 (3.3)	9 (2.9)	0.807
Duration of cirrhosis (years)	4.6 ± 3.1	4.7 ± 3.6	0.689
Commodities, *n* (%)	38 (25.2)	72 (23.2)	0.633
White blood cell (× 10^9^/L)	3.6 (2.3–5.3)	4.0 (2.7–5.8)	0.018
Red blood cell (× 10^9^/L)	3.2 (2.7–3.6)	3.3 (2.8–3.7)	0.325
Hemoglobin (g/L)	97.0 (80.5–111.0)	103.0 (87.0–118.0)	0.021
Platelet count (× 10^9^/L)	69.0 (45.5–97.0)	66.0 (43.0–104.0)	0.700
PT (s)	16.7 (14.7–18.8)	17.0 (14.9–19.0)	0.368
International normalized ratio	1.39 (1.2–1.6)	1.4 (1.3–1.7)	0.053
ALT (IU/L)	22.1 (15.0–35.6)	31.0 (20.0–52.0)	<0.001
AST (IU/L)	37.0 (26.4–52.5)	53.0 (35.0–85.0)	<0.001
Albumin (g/L)	30.9 (28.0–33.5)	29.1 (26.5–32.8)	<0.001
Total bilirubin (µmol/L)	28.6 (17.9–41.4)	34.0 (20.8–75.1)	<0.001
Creatinine (µmol/L)	78.0 (64.2–97.0)	80.0 (65.5–102.0)	0.539
BUN (mmol/L)	5.7 (4.1–7.4)	5.9 (4.2–8.3)	0.488
Sodium (mmol/L)	138.0 (135.0–140.0)	138.0 (135.0–140.0)	0.654
Child–Pugh score	9.0 (8.0–9.5)	9.0 (8.0–11.0)	<0.001
Child–Pugh class (B/C)	**–**	**–**	<0.001
B (7–9)	113 (74.8)	170 (54.7)	–
C (10–13)	38 (25.2)	141 (45.3)	–
MELD score (points)	12.5 (10.6–15.7)	14.4 (11.0–19.0)	<0.001
MELD score, *n* (%)	**–**	**–**	<0.001
≤11	48 (31.8)	84 (27.0)	**–**
11–19	87 (57.6)	144 (46.3)	**–**
≥19	16 (10.6)	83 (26.7)	**–**
FIPS score	0.11 (−0.48 to 0.64)	0.17 (−0.42 to 0.70)	<0.001
FIPS score, *n* (%)	**–**	**–**	0.060
FIPS ≥ 0.92	51 (33.8)	79 (25.4)	**–**
FIPS < 0.92	100 (66.2)	232 (74.6)	**–**

ALT, alanine aminotransferase; AST, aspartate aminotransferase; BUN, blood urea nitrogen; LVP + A, repetitive large-volume paracentesis plus albumin; MELD, model for end-stage liver disease; TIPS, transjugular intrahepatic portosystemic shunt.

Values are expressed as mean ± standard deviation or number (%).

The median follow-up time was 36.0 ± 1.5 months for the whole cohort, 36.0 ± 2.1 months for the TIPS group, and 36.0 ± 2.0 months for the LVP + A group. During this period, 10 patients (2.3%) (8 in the TIPS group and 2 in the LVP + A group) were lost to follow-up within 1 year.

### Mortality

**Figure 1.**
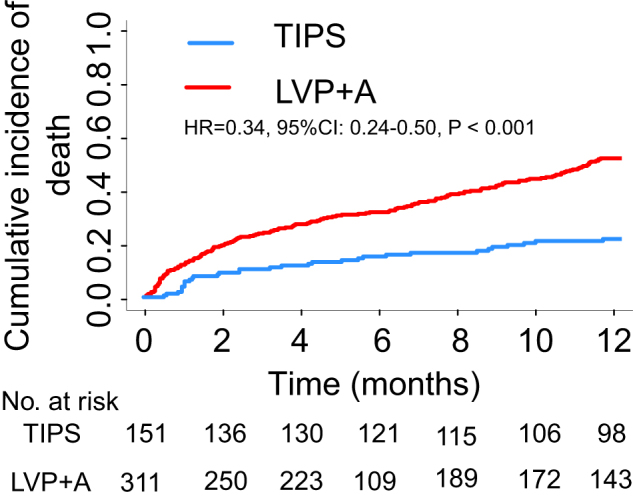
Cumulative incidence of death at 1 year according to treatment groups in the entire population. TIPS, transjugular intrahepatic portosystemic shunt; LVP + A, repetitive large-volume paracentesis plus albumin.

**Figure 2.**
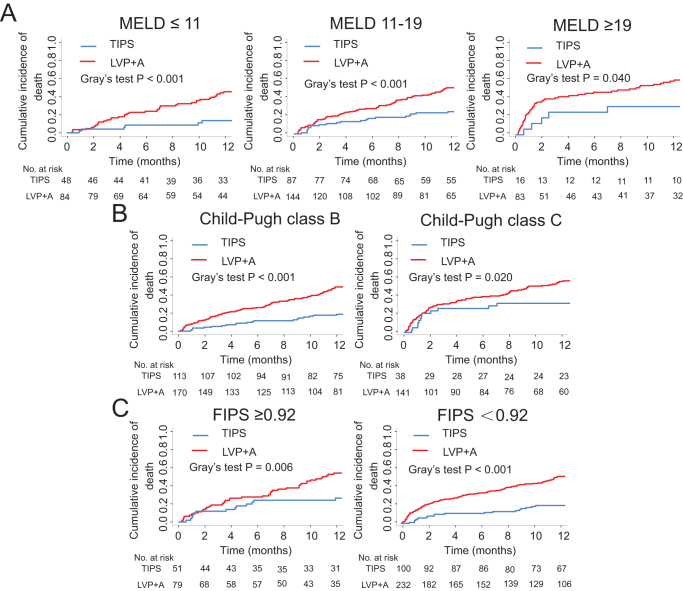
Cumulative incidence of death at 1 year in the TIPS group vs. LVP + A group stratified according to (A) MELD 11–19 rules, (B) Child–Pugh class, (C) FIPS score based on competing risk approach (the Fine and Gray method) with liver transplantation being the competing events.

**Figure 3.**
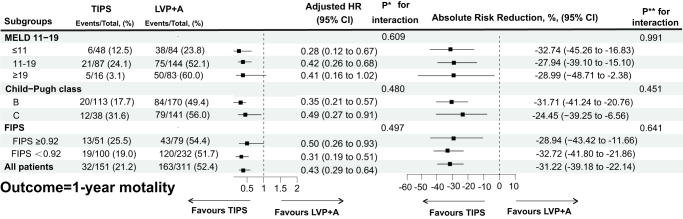
Event rate, adjusted HRs, and the absolute risk reduction for the 1-year mortality by risk categories and treatment groups.

In the entire cohort, 195 patients (42.2%) died within 1 year. The causes of death are summarized in Table 2. Compared with the LVP + A group, the cumulative incidences of death were significantly lower in the TIPS group at 1 year (21.2% vs. 52.4%, *P* < 0.001) (Fig. 1). After adjusting for potential confounders, the risk of death with TIPS was reduced by 57% at 1 year (adjusted HR = 0.43; 95% CI: 0.29–0.64; *P* < 0.001) (Table 3). This finding was confirmed by the analyses of the treatment effect in subgroups of patients according to the risk category of three classification rules (Figs. 2 and 3).

**Table 2:** Summary of outcome measurements.

Outcome	TIPS (*n* = 151)			LVP + A (*n* = 311)		
	6 months	1 year	2 years	6 months	1 year	2 years
Motality, *n* (%)	23 (15.2)	32 (21.2)	37 (24.5)	100 (32.2)	163 (52.4)	179 (57.6)
ACLF	8 (5.30)	9 (5.96)	9 (5.96)	22 (7.07)	39 (12.5)	43 (13.8)
Gastrointestinal bleeding	6 (3.97)	8 (5.30)	9 (5.96)	17 (5.47)	24 (7.72)	28 (9.00)
Multiple organ failure	1 (0.67)	1 (0.67)	2 (1.32)	9 (2.89)	10 (3.22)	10 (3.22)
Pneumonia	1 (0.67)	1 (0.67)	1 (0.67)	2 (0.64)	4 (1.29)	4 (1.29)
Hepatocellular carcinoma	0	0	0	0	0	1 (0.3)
Ascites	2 (1.32)	2 (1.32)	2 (1.32)	23 (7.40)	31 (9.97)	34 (10.9)
OHE	2 (1.32)	2 (1.32)	4 (2.6)	6 (1.93)	9 (2.89)	9 (2.89)
Respiratory failure	0	1 (0.67)	1 (0.67)	2 (0.64)	7 (2.25)	11 (3.54)
Septic shock secondary to SBP	2 (1.32)	2 (1.32)	2 (1.32)	7 (2.25)	12 (1.61)	12 (1.61)
Systemic infection	1 (0.67)	2 (1.32)	2 (1.32)	7 (2.25)	12 (1.61)	12 (1.61)
Heart failure with atrial fibrillation	0	1 (0.67)	2 (0.67)	3 (0.96)	5 (1.61)	5 (1.61)
Other reasons (found dead at home)	0	3 (1.99)	3 (1.99)	4 (1.29)	10 (3.22)	10 (3.22)
Main clinical outcomes	–	–	–	–	–	–
Ascite recurrence, *n* (%)	21 (13.9)	27 (17.9)	33 (21.9)	311 (100.0)	311 (100.0)	311 (100.0)
Over hepatic encephalopathy, *n* (%)	34 (22.5)	40 (26.5)	42 (27.8)	17 (5.5)	20 (6.4)	23 (7.4)
Uncontrolled bleeding	11 (7.3)	16 (10.6)	18 (11.9)	59 (19.0)	83 (26.7)	95 (30.5)
Hepatorenal syndrome	0	1 (0.67)	1 (0.67)	0	2 (0.64)	3 (0.96)
Hepatomyelopathy	0	2 (1.32)	2 (1.32)	0	2 (0.64)	3 (0.96)

LVP + A, repetitive large-volume paracentesis plus albumin; TIPS, transjugular intrahepatic portosystemic shunt.

**Table 3:** Univariate and multivariate analyses of factors associated with 1-year mortality in the entire cohort (*n* = 462).

Variables	Uultivariate analysis		Multivariate analysis	
	HR (95% CI)	*P* values	HR (95% CI)	*P* values
Treatment (TIPS vs. LVP + A)	0.34 (0.24–0.50)	<0.001	0.43 (0.29–0.64)	<0.001
Age (per year increase)	1.02 (1.01–1.04)	<0.001	1.02 (1.01–1.04)	<0.001
HBV-DNA detectable (yes vs. no)	2.46 (1.84–3.29)	<0.001	2.78 (2.02–3.83)	<0.001
WBC (per 10^9^ increase)	1.07 (1.04–1.11)	<0.001	1.06 (1.01–1.10)	0.011
Serum albumin (per g/L increase)	0.96 (0.93–0.98)	0.002	–	–
CRE (per µmol/L increase)	1.21 (1.10–1.33)	<0.001	1.19 (1.08–1.31)	<0.001
BUN (per mg/dL increase)	1.06 (1.03–1.08)	<0.001	1.04 (1.00–1.18)	0.042
Serum sodium (per mg/dL increase)	0.96 (0.93–0.99)	0.005	0.97 (0.94–1.00)	0.051
Child–Pugh score (per point increase)	1.19 (1.09–1.31)	<0.001	–	–
MELD score (per point increase)	1.07 (1.05–1.10)	<0.001	–	–
FIPS score (per point increase)	2.25 (1.22–4.15)	<0.001	–	–
Previous variceal bleeding (yes vs. no)	3.81 (2.76–5.25)	<0.001	3.43 (2.47–4.78)	<0.001

Specifically, according to MELD 11–19 rules, the TIPS group had a significantly lower cumulative incidence of death in the MELD ≤ 11 category (12.5% vs. 23.8%, *P* < 0.001), MELD 12–18 category (24.1% vs. 52.1%, *P* < 0.001), MELD ≥ 19 category (3.1% vs. 60.0%, *P* = 0.040) compared with the medical group (Fig. 2A), Notably, the sample size of patients in the subgroup with MELD ≥19 was relatively small, which may result in insufficient statistical power of the results. This pattern persisted after adjusting for potential confounders, with the adjusted HRs (95%CI) being 0.28 (0.12–0.67), 0.42 (0.26–0.68), and 0.41 (0.16–1.02) at 1 year, respectively (Fig. 3 and Supplemental Digital Content Table 1, available at: http://links.lww.com/JS9/G587).

Similar patterns emerged when patients were stratified according to their Child–Pugh class. In Child–Pugh C class patients, the cumulative incidences of death were significantly lower in the TIPS group at 1 year (31.6% vs. 56.0%, *P* = 0.020). In Child–Pugh B class patients, the cumulative incidences of death were significantly lower in the TIPS group (17.7% vs. 49.4%, *P* < 0.001) (Fig. 2B). This pattern was not altered after adjusting for potential confounders, with the adjusted HRs (95%CI) being 0.35 (0.21–0.57), 0.49 (0.27–0.91) at 1 year, respectively (Fig. 3 and Supplemental Digital Content Table 1, available at: http://links.lww.com/JS9/G587).

Per the FIPS score, significantly lower cumulative incidences of death in the TIPS group were observed in the FIPS ≥0.92 category (25.5% vs. 54.4%, *P* = 0.006) and FIPS < 0.92 category (19.0% vs. 51.7%, *P* < 0.001) (Fig. 2C). Similar results were observed after adjusting for potential confounders, with the adjusted HRs (95%CI) being 0.50 (0.26–0.93), 0.31 (0.19–0.51) at 1 year, respectively (Fig. 3 and Supplemental Digital Content Table 1, available at: http://links.lww.com/JS9/G587).

### Liver and kidney functions

**Figure 4.**
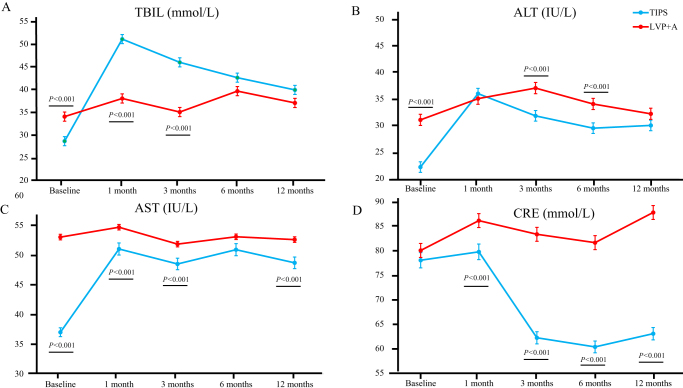
The changes of liver and kidney functions over 1 year.

As shown in Figure 4 and Table 4, the TIPS group had increased bilirubin levels at 1 month to 6 months (*P* < 0.001). After 12 months, the ALT of the TIPS group and LVP + A group were 30.0 U/L (16.3–45.6) and 32.0 U/L (20.8–68.1), respectively (*P* = 0.462), and AST were 48.7 U/L (33.7–76.5) and 52.6 U/L (30.8–78.2) (*P* < 0.001), respectively. The TIPS group had lower creatinine at these times, whereas at 1 month to 1 year after TIPS, there was significant difference in creatinine between the two groups (*P* < 0.001).

**Table 4:** The changes of liver and kidney functions over 1 year.

	TIPS group (*N* = 151)	LVP + A group (*N* = 311)	*P*
Baseline	–	–	–
ALT (IU/L)	22.1 (15.0–35.6)	31.0 (20.0–52.0)	<0.001
AST (IU/L)	37.0 (26.4–52.5)	53.0 (35.0–85.0)	<0.001
Total bilirubin (µmol/L)	28.6 (17.9–41.4)	34.0 (20.8–75.1)	<0.001
Creatinine (µmol/L)	78.0 (64.2–97.0)	80.0 (65.5–102.0)	0.539
1 month	–	–	–
ALT (IU/L)	35.9 (20.1–65.6)	35.0 (18.0–62.0)	0.852
AST (IU/L)	51.0 (32.4–80.3)	54.7 (34.0–89.7)	<0.001
Total bilirubin (µmol/L)	51.1 (37.9–68.9)	38.0 (26.8–55.1)	<0.001
Creatinine (µmol/L)	79.7 (65.4–98.6)	86.1 (75.2–110.0)	<0.001
3 months	–	–	–
ALT (IU/L)	31.8 (21.4–52.5)	37.0 (25.0–56.0)	<0.001
AST (IU/L)	48.5 (31.2–73.6)	51.8 (30.0–79.5)	<0.001
Total bilirubin (µmol/L)	46.0 (30.5–72.0)	35.0 (25.4–56.5)	<0.001
Creatinine (µmol/L)	62.1 (52.7–86.5)	83.3 (71.4–102.5)	<0.001
6 months	–	–	–
ALT (IU/L)	29.0 (18.9–44.4)	34.0 (24.8–55.1)	<0.001
AST (IU/L)	50.9 (31.6–72.8)	53.0 (31.4–76.5)	0.032
Total bilirubin (µmol/L)	42.6 (28.4–78.5)	39.6 (26.5–62.5)	0.012
Creatinine (µmol/L)	60.3 (51.4–80.5)	81.6 (70.0–97.5)	<0.001
12 months	–	–	–
ALT (IU/L)	30.0 (16.3–45.6)	32.0 (20.8–68.1)	0.462
AST (IU/L)	48.7 (33.7–76.5)	52.6 (30.8–78.2)	<0.001
Total bilirubin (µmol/L)	39.9 (26.4–58.5)	37.0 (24.4–56.4)	0.729
Creatinine (µmol/L)	63.3 (52.1–82.6)	87.8 (76.5–112.5)	<0.001

### Recurrence of ascites

During the 1-year follow-up, 62 (41%) patients in the TIPS group achieved complete resolution of ascites and did not require any further diuretic therapy, compared to none in the LVP + A group, where all patients required continued diuretic use alongside paracentesis (*P* < 0.001). This indicates a more definitive control of ascites with TIPS. The use of spironolactone and furosemide during this period decreased from 106 ± 40 to 85 ± 33 mg and 45 ± 25 to 34 ± 21 mg per day in the TIPS group and remained almost unchanged in the LVP group (128 ± 37 and 56 ± 27 mg per day at baseline and 133 ± 43 and 49 ± 30 mg per day at 1 year; *P* < 0.001 for the difference between groups). In the TIPS group, seven patients developed shunt dysfunction resulting in persistence of ascites after the procedure, and were treated successfully by insertion of a second stent. As a whole, the cumulative incidence of recurrent ascites requiring paracentesis was significantly decreased in the TIPS group at 1 year (17.9% vs. 100%, *P* < 0.001) compared with the LVP + A group (Table 2).

### Overt HE

There are significant differences in the cumulative incidence of OHE at 1 year (TIPS, 26.5% vs. LVP + A, 6.4%; *P* < 0.001) (Table 2). These results were not altered after adjusting for the potential confounders (Supplemental Digital Content Table 2, available at: http://links.lww.com/JS9/G477). In stratification analyses, the RRR in OHE did not significantly differ among the risk categories. However, the absolute risk reductions (ARRs) of OHE was more pronounced in high-risk patients. The ARRs at 1 year were 8.93%, 22.49%, and 46.61% in model for end-stage liver disease (MELD) ≤ 11, 11–19 and ≥ 19 patients and were 16.83% and 31.67% in Child–Pugh B and C patients, respectively (interaction tests, *P* < 0.001 for both criteria) (Supplemental Digital Content Figure 2, available at: http://links.lww.com/JS9/G475).

### Uncontrolled bleeding

The cumulative incidence of uncontrolled bleeding was lower in the TIPS group at 1 year (10.6% vs. 26.7%, *P* < 0.001) compared with the LVP + A group. This pattern was not altered after adjusting for potential confounders (Supplemental Digital Content Table 3, available at: http://links.lww.com/JS9/G478). In the stratification analysis, the ARR of uncontrolled bleeding with TIPS was more pronounced in the lower risk patients (Supplemental Digital Content Figure 3, available at: http://links.lww.com/JS9/G475).

### Sensitivity analysis

Imputation of missing data and imbalance in data distribution between groups are important causes of differences in statistical results. After deletion of missing values, the baseline characteristics were shown in Supplemental Digital Content Table 4, available at: http://links.lww.com/JS9/G479, the statistical results of motality, ascite recurrence, over HE and uncontrolled bleeding were robust in TIPS versus LVP + A comparisons (Supplemental Digital Content Figure 4, available at: http://links.lww.com/JS9/G475 and Supplemental Digital Content table 5, available at: http://links.lww.com/JS9/G480 and Supplemental Digital Content table 6, available at: http://links.lww.com/JS9/G481). Additionally, regarding the sensitivity analysis performed via PSM (Supplemental Digital Content Table 7, available at: http://links.lww.com/JS9/G482), the results remained stable for the comparison between TIPS and LVP + A, with the findings presented in Supplemental Digital Content Figure 5, available at: http://links.lww.com/JS9/G475 and Supplemental Digital Content Table 8, available at: http://links.lww.com/JS9/G483 and Supplemental Digital Content Table 9, available at: http://links.lww.com/JS9/G484.

## Discussion

In this multicenter observational study, we showed that TIPS was associated with improved survival, decreased uncontrolled bleeding, and recurrent ascites, with increasing the risk of developing OHE. This study has several strengths. First, we included a wide range of real-world patients with recurrent ascites from multiple centers, providing a more comprehensive view of the survival benefits of TIPS for patients. Second, our study validated the benefit of TIPS in patients with recurrent ascites before they progress to RA. The results showed that TIPS significantly improved patients survival compared with LVP + A. Third, although TIPS with covered stents increased the risk of HE, it could efficiently control the recurrence of ascites and PHT-related bleeding.

Current guidelines recommend TIPS for patients with recurrent ascites to improve patient survival[21]. The definition of recurrent ascites by ICA does not have special diagnostic criteria such as RA, but it is determined by the number of recurrent massive ascites within a year[5]. Bureau *et al* included patients with recurrent ascites who had at least two LVPs in the past 3 weeks and fewer than six LVPs in 3 months. They concluded that PTFE-covered stent TIPS could improve survival in patients with recurrent ascites without increasing the incidence of HE[20]. Shen *et al* defined TIPS for such patients as early TIPS[29]. However, they did not discuss the outcomes in relation to enhanced survival and prognosis of associated complications in patients with recurrent ascites. Another case–control study, exclusively involving ePFTE-TIPS, revealed that TIPS improves survival in patients with RA after 1 year but loses its benefits beyond that time[30].

Patients who received TIPS with covered stents survived longer than those who received repeated LVP + A therapy, with a cumulative incidence of death at 1 year (21.2% vs. 52.4%). These findings are consistent with those of two meta-analyses, suggesting that TIPS could improve survival in patients with recurrent ascites^[14,15].^ Given the heterogeneity in the clinical response to TIPS observed in RCTs, clinicians are often reluctant to deploy TIPS as a first-line therapy for RA[31]. For recurrent ascites, the evidence consistently suggests that TIPS could improve the survival of these patients without transplantation^[14,15,20]^. Currently, no consensus exists on the timing of TIPS implantation. Many international guidelines, including the ICA (1996), EASL (2010), and American Association for the Study of Liver Diseases (2021) guidelines, regard TIPS as a second-line treatment when LVP treatment is too frequent or ineffective^[4,22,32]^. The North American TIPS guidelines suggest that patients who do not meet the strict diagnostic criteria for RA require LVP >3 times/year after receiving the maximum dose of diuretics to be considered for TIPS treatment[33]. The 2022 Consensus on PHT (Baveno VII) proposes that, regardless of the presence of varicose veins or varicose bleeding history, patients with recurrent ascites (requiring LVP for 1 year, regardless of the number of times) may be considered for TIPS therapy[34]. Chinese guidelines on the management of ascites state that there is no consensus on whether early TIPS should be used for the treatment of RA[35]. Therefore, our results highlight the importance of TIPS implantation in patients with severe ascites, rather than waiting until paracentesis is ineffective. When patients with recurrent ascites require frequent LVP treatment, TIPS therapy should be considered as soon as possible. Otherwise, when the disease progresses to an advanced stage and liver and kidney functions deteriorate, not only may surgical contraindications arise but the degree of postoperative benefit also becomes limited.

TIPS is significantly better than LVPs in controlling the recurrence of ascites, which is consistent with the results of previous RCTs.^[8–13,20]^ Since implantation of covered stents could reduce shunt dysfunction more than implantation of bare stents, better control of ascites could be achieved^[36,37]^. Therefore, we included all the patients with covered stents to eliminate confounding factors. Our results showed that TIPS can significantly decreased the recurrence of ascites at 1 year (17.9% vs. 100%).

HE is a major complication of TIPS, especially in patients using bare stents, which can occur in up to 50% of them^[38–40]^. According to a recent RCT, compared with a 10 mm bracket, the use of an 8-mm PTFE-covered stent significantly reduced the incidence of HE to 18%[41]. The primary stent type utilized in our study was the 8-mm PTFE-covered stent, and the final occurrence of HE in the TIPS group was 26.5% at 1 year, aligning with findings from previous research^[12,20]^. A recent multicenter, non-inferiority observational study aimed to assess the 30-month mortality rate in patients who underwent TIPS procedures, with and without OHE. The findings indicated that the occurrence of OHE following TIPS does not elevate the mortality risk, regardless of the underlying reason for the procedure. This finding alleviates concerns regarding the weight of this complication after TIPS[42]. Furthermore, our subgroup analysis suggests that the survival benefit of TIPS is more pronounced in patients with preserved hepatic reserve (MELD < 19). This supports the strategy of considering TIPS intervention not only before ascites control becomes difficult but also before the progression to advanced liver dysfunction. While current guidelines recommend TIPS as a second-line treatment, an RCT study published in Gastroenterology in 2017 provides us with insights: for certain patients, undergoing TIPS earlier may yield survival benefits, and TIPS could potentially serve as a first-line treatment option for such patients[20]. Our study included patients who received TIPS at a relatively early stage based on multidisciplinary assessment. This result represents a shift in clinical practice, rather than strict adherence to previous guidelines; however, further RCTs are still needed to validate the efficacy of early TIPS use in appropriate patients.

This study had some limitations. First, we did not routinely measure the mean portal vein pressure gradient in patients with LVP + A, which made it impossible to compare results with those in the TIPS group. Second, because the shortage of available donor organs limits the potential number of organ transplantations in China[43], the LT rate in our cohort was very low. Third, although our study focused on clinical outcomes, future prospective studies incorporating patient-reported outcomes, such as health-related quality of life, would provide a more comprehensive evaluation of the benefits of TIPS versus repeated paracentesis. Fourth, as a retrospective observational study, inherent selection bias, residual confoundings and patients’ loss to follow-up remain unavoidable. The allocation of patients to TIPS or LVP + A groups was primarily determined by individual center protocols and treating physicians’ clinical judgment – this decision-making process may have favored TIPS for patients with more stable clinical conditions. To overcome this problem, we conducted multivariate Cox analyses and PSM to control for key measured confounders. However, residual confounding due to unmeasured or unrecorded factors (such as subtle differences in nutritional status, specific center-level practices, or socioeconomic factors) persists even after these adjustments. These unmeasured factors could potentially exaggerate or attenuate the observed survival benefit of TIPS. Therefore, while our findings provide strong real-world evidence, our results should be interpreted with caution, and the observed associations between TIPS and improved outcomes need to be validated in well-designed prospective RCTs that can prospectively define inclusion criteria, standardize treatment allocation, ensure the long-term follow-up of patients and systematically collect comprehensive data on both measured and potential unmeasured confounders.

In conclusion, compared with LVP + A, TIPS could improve the liver and kidney functions and survival of patients, and may be considered a first-line treatment option for patients with recurrent ascites.

## Data Availability

This article is available upon reasonable request.

## Research registration unique identifying number (UIN)

This study has been registered on ClinicalTrials.gov with the unique identifier NCT06196723.
